# Prognostic impact of the cumulative dose and dose intensity of everolimus in patients with pancreatic neuroendocrine tumors

**DOI:** 10.1002/cam4.1028

**Published:** 2017-05-25

**Authors:** Rossana Berardi, Mariangela Torniai, Sara Pusceddu, Francesca Spada, Toni Ibrahim, Maria Pia Brizzi, Lorenzo Antonuzzo, Piero Ferolla, Francesco Panzuto, Nicola Silvestris, Stefano Partelli, Benedetta Ferretti, Federica Freddari, Calogero Gucciardino, Enrica Testa, Laura Concas, Sabina Murgioni, Alberto Bongiovanni, Clizia Zichi, Nada Riva, Maria Rinzivillo, Oronzo Brunetti, Lucio Giustini, Francesco Di Costanzo, Gianfranco Delle Fave, Nicola Fazio, Filippo De Braud, Massimo Falconi, Stefano Cascinu

**Affiliations:** ^1^ Clinica di Oncologia Medica Università Politecnica delle Marche AOU Ospedali Riuniti di Ancona Italy; ^2^ Medicina Oncologica 1 ENETS Center of excellence Fondazione IRCCS Istituto Tumori Milano Italy; ^3^ Unità di Oncologia Medica Gastrointestinale e Tumori Neuroendocrini (Unit of Gastrointestinal Medical Oncology and Neuroendocrine Tumors) IEO Istituto Europeo di Oncologia Milano Italy; ^4^ Osteoncology and Rare Tumors Center Istituto Scientifico Romagnolo per lo Studio e la Cura dei Tumori (IRST) IRCCS Meldola Italy; ^5^ Oncologia Medica A.O.U. San Luigi Orbassano (TO) Italy; ^6^ SC di Oncologia Medica Azienda Opedaliero‐Universitaria Careggi Firenze Italy; ^7^ Doctorate Course in Genetics Oncology and Clinical Medicine University of Siena Siena Italy; ^8^ Multidisciplinary NET Group Umbria Regional Cancer Network Perugia Italy; ^9^ Digestive and Liver Disease Sapienza University of Rome Sant'Andrea Hospital Rome Italy; ^10^ Medical Oncology Unit National Cancer Institute Giovanni Paolo II Bari Italy; ^11^ Chirurgia del Pancreas Università Politecnica delle Marche AOU Ospedali Riuniti di Ancona Italy; ^12^ Chirurgia del Pancreas Ospedale San Raffaele IRCCS Università Vita e Salute Milano Italy; ^13^ Oncologia Medica Ospedale di San Severino San Severino Marche (MC) Italy; ^14^ Oncologia Medica Ospedale di Senigallia Senigallia Italy; ^15^ Oncologia Medica Ospedale di Fermo Fermo Italy; ^16^ Oncologia Medica Ospedale di Urbino Urbino Italy; ^17^ Oncologia Medica Università di Modena e Reggio Emilia Modena Italy

**Keywords:** Everolimus, mTOR inhibitor, pancreatic neuroendocrine tumors, targeted therapy

## Abstract

The aim of this work is to assess if cumulative dose (CD) and dose intensity (DI) of everolimus may affect survival of advanced pancreatic neuroendocrine tumors (PNETs) patients. One hundred and sixteen patients (62 males and 54 females, median age 55 years) with advanced PNETs were treated with everolimus for ≥3 months. According to a Receiver operating characteristics (ROC) analysis, patients were stratified into two groups, with CD ≤ 3000 mg (Group A; *n* = 68) and CD > 3000 mg (Group B; *n* = 48). The response rate and toxicity were comparable in the two groups. However, patients in group A experienced more dose modifications than patients in group B. Median OS was 24 months in Group A while in Group B it was not reached (HR: 26.9; 95% CI: 11.0–76.7; *P* < 0.0001). Patients who maintained a DI higher than 9 mg/day experienced a significantly longer OS and experienced a trend to higher response rate. Overall, our study results showed that both CD and DI of everolimus play a prognostic role for patients with advanced PNETs treated with everolimus. This should prompt efforts to continue everolimus administration in responsive patients up to at least 3000 mg despite delays or temporary interruptions.

## Introduction

Pancreatic neuroendocrine tumors (PNETs) are still considered a rare disease which accounts for approximately 10% of all cases of pancreatic cancer 1. Nevertheless, the increasing incidence and prevalence of PNETs observed in the last four decades 2, together with the frequent delay in diagnosis 3, 4, have led to increasing interest in PNETs, with major advances in their treatment and management 5.

Among these advances, the elucidation of the high expression and activity of mammalian target of rapamycin (mTOR) in PNETs has led to the recognition of mTOR as an important therapeutic target 6. The mTOR serine/threonine protein kinase pathway plays a key role in cell growth and proliferation, angiogenesis, and nutrient uptake by increasing protein synthesis 7, 8. A pathological activation of this pathway has been reported in a variety of cancers, including PNETs, with a primary role in tumorigenesis 9; therefore, strategies aimed at interfering with mTOR function could represent effective approaches in the targeted therapy of PNETs.

Based on the results of the phase III RADIANT‐3 trial 10, the oral mTOR inhibitor everolimus has become an established recommended standard therapy for patients with advanced PNETs. Although everolimus exerts a very selective action on a specific molecular target, this drug may be associated with a number of adverse effects, included stomatitis, rash, fatigue, pneumonitis, and metabolic alterations mainly represented by hyperlipidemia. Other common events include abdominal pain, nausea and/or vomiting, anemia, increased serum creatinine level, liver function test abnormalities, dizziness, headache, and epistaxis 11, 12, 13. These adverse effects frequently lead to modify the dosage by drug delay and/or reduction of dose, with a significant impact on cumulative dose (CD) and dose intensity (DI).

In this study, we aimed to evaluate the effect of CD and DI—defined as the total amount of everolimus taken by the patient despite delay or dose reductions and as drug dose delivered per time unit, respectively—on survival of patients treated with everolimus for unresectable or metastatic PNETs.

## Patients and Methods

### Patients

In this nonrandomized retrospective study, we included all the consecutive patients with advanced (unresectable or metastatic) PNETs treated with everolimus in 14 Italian institutions between December 2009 and December 2015. All the data were prospectively collected and retrospectively analyzed for the purpose of the study. Recorded patient characteristics and clinical features included the following: gender, age, neoplastic grading (by the Ki67 labeling index of the WHO 2010 classification 14) and histological features, immunohistochemical staining for general neuroendocrine markers, stage of disease (T, N, M) at the time of the diagnosis (according to the TNM Seventh edition (2010) 15), and data regarding all the treatments received by the patients.

### Study design and treatment regimens

Everolimus was administered orally at a dose of 10 mg once daily in all patients with the exception of 11 patients who started with 5 mg daily. Treatment was continued until progression of the disease, development of unacceptable toxicity or death. Doses were delayed or reduced if the patient experienced clinically relevant adverse events, according to standard guidelines and clinical practice.

Follow‐up comprised regular history‐taking, physical examination, and laboratory assessment (hematologic and serum chemical measurement) every week, and imaging studies through computed tomography (CT) or magnetic resonance imaging (MRI) every 8–12 weeks. Response to therapy was assessed according to the RECIST 1.1 (Response Evaluation Criteria In Solid Tumors) 16. All assessments were confirmed at a central level.

CD was defined as the total amount of everolimus taken by the patient despite delay or dose reductions; DI was defined as everolimus dose delivered per time unit (mg/day) taken by the patient divided by the days of therapy (including temporary interruptions).

### Statistical analysis

Overall survival (OS) was defined as the interval between the initiation of everolimus‐based treatment to death from any cause or to the last follow‐up visit. Patients who were not reported as dead at the time of the analysis were censored at the date they were last known to be alive. Progression‐free survival (PFS) was defined as the interval between the initiation of everolimus treatment and disease progression or death. Survival distribution was estimated by the Kaplan–Meier method. Significant differences in probability of surviving between the strata were evaluated by log‐rank test. Receiver operating characteristics (ROC) curve analysis was performed to determine a cut off value for the CD of everolimus. The Cox multivariate proportional hazard regression model was used to evaluate the effects of a number of factors on OS. A level of 0.05 was chosen to assess the statistical significance. Statistical analyses were performed using MedCalc version 11.4.4.0 (MedCalc Software, Broekstraat 52, 9030 Mariakerke, Belgium).

## Results

In total, 116 patients were evaluated. Median age was 55 years old (range 19–89), and male/female ratio was 62/54. As for grading determination, grading showed G2 predominance (25% G1 and 75% G2). Twenty‐eight patients died during follow‐up (Table 1).

**Table 1:** Clinical‐pathological characteristics, surgical, and medical history of the enrolled patients

	*N* (%)
*Patients*	116 (100.0)
Gender	
Male	62 (53.5)
Female	54 (46.5)
*Age — year median (range)*	55 (19–89)
Histological grading	
Ki67 < 3% (G1)	29 (25)
Ki67 3–20% (G2)	87 (75)
Stage at the initial diagnosis	
Localized	6 (5.2)
Locally advanced	18 (15.5)
Metastatic	92 (79.3)
Surgery	
Not performed	56 (48.3)
Performed	60 (51.7)
Debulking surgery and/or metastasectomy	39 (33.6)
Radical surgery	21 (18.1)
SSa	
Not performed	8 (6.9)
Performed	108 (93.1)
PRRT	
Not performed	62 (53.5)
Performed	54 (46.5)
Death	
No	88 (75.9)
Yes	28 (24.1)

All patients received everolimus for advanced/metastatic PNETs. With respect to previous history, 110 patients presented with locally advanced or metastatic disease at diagnosis and 56 of them (48.3%) did not undergo surgical resection. Among the remaining 60 patients (51.7%), 39 subjects (33.6%) had undergone debulking surgery and/or metastasectomy, but presented with recurrent/progressive disease when everolimus was started. Previous radical resection was performed in 21 patients (18.1%), all of them relapsed with liver metastases. Loco‐regional therapies were performed in 23 patients: transarterial chemoembolization as loco‐regional therapy in 18 patients, radiofrequency in the remaining 5. Almost all patients (93.1%) received somatostatin analogs and 54 patients received peptide receptor radionuclide therapy (PRRT) (Table 1). Everolimus was administrated together with somatostatin analogs (SSas) in 82 patients, while 34 patients received the target therapy alone.

Table 2 presents the previous lines of therapies and summarizes the outcomes of everolimus therapy. All patients received everolimus for at least 3 months and only in twenty‐two cases (19.0%) as first‐line therapy.

**Table 2:** Details of everolimus therapy in the overall population

	*N* (%)
Everolimus	116 (100.0)
Patients treated with first‐line everolimus (administrated together with SSa in 17 patients)	22 (19.0)
*Patients treated with second‐line everolimus (administrated together with SSa in 32 patients)*	38 (32.8)
Prior treatments	
SSa	32 (84.2)
PRRT	5 (13.2)
CHT	1 (2.6)
*Cisplatin+5‐Fluorouracil*	1 (100.0)
Patients treated with third‐line everolimus (*administrated together with SSa in 26 patients)	38 (32.8)
Prior treatments	
SSa	31 (81.6)
PRRT	24 (63.2)
Sunitinib	3 (7.9)
IFN	1 (2.6)
CHT	13 (34.2)
Cisplatin+Etoposide → PRRT	2 (15.3)
Cisplatin+Etoposide → Topotecan	1 (7.7)
Capecitabine+Oxaliplatin → SSa	1 (7.7)
Capecitabine → SSa	1 (7.7)
Gemcitabine+Oxaliplatin → Capecitabine	1 (7.7)
SSa → Capecitabine+Temozolomide	1 (7.7)
Cisplatin+Etoposide →SSa	1 (7.7)
SSa → Cisplatin+Etoposide	1 (7.7)
SSa → Capecitabine+Oxaliplatin	1 (7.7)
5‐Fluorouracil → Capecitabine	1 (7.7)
Capecitabine → PRRT	1 (7.7)
Capecitabine+Oxaliplatin → Capecitabine	1 (7.7)
*Patients treated with fourth‐line everolimus (administrated together with SSa in 4 patients)*	12 (10.3)
Prior treatments	
SSa	10 (83.3)
PRRT	9 (75.0)
CHT	12 (100.0)
SSa → PRRT → Capecitabine	2 (17.0)
SSa → PRRT → Capecitabine	1 (8.3)
SSa → Capecitabine → Temozolomide	1 (8.3)
SSa → 5‐Fluorouracil+Epiribicin+Temozolomide →PRRT	1 (8.3)
Cisplatin+Etoposide → Temozolomide → Paclitaxel	1 (8.3)
Carboplatin+Etoposide → Oxaliplatin+5‐Fluorouracil → 5‐Fluorouracil+Irinotecan	1 (8.3)
Cisplatin+Etoposide → SSa → PRRT	1 (8.3)
SSa → Cisplatin+Etoposide → PRRT	1 (8.3)
SSa → PRRT → Capecitabine+Bevacizumab	1 (8.3)
SSa → Capecitabine → PRRT	1 (8.3)
Oxaliplatin+Capecitabine → SSa → PRRT	1 (8.3)
Patients treated with fifth‐line everolimus (*administrated together with SSa in 1 patient)	6 (5.1)
Prior treatments	6 (100.0)
SSa	6 (100.0)
PRRT	6 (100.0)
CHT	1 (16.6)
SSa → 5‐Fluorouracil → PRRT → Capecitabine	1 (16.6)
SSa → Cisplatin+Etoposide → PRRT → Capecitabine	1 (16.6)
SSa → Cisplatin+Etoposide → PRRT → Cisplatin+Etoposide	1 (16.6)
Carboplatin+Etoposide → PRRT → PRRT → Capecitabine	1 (16.6)
Gemcitabine → Capecitabine+Oxaliplatin → SSa → PRRT	1 (16.6)
SSa → Capecitabine+Temozolomide → PRRT → 5‐Fluorouracil+Oxaliplatin	1 (16.6)
Dose delay	
No	39 (33.6)
Yes	77 (66.4)
Days of delay in patients with everolimus interruption	
Median	28
Range	5–279
Dose reduction	
No	91 (78.4)
Yes	25 (21.6)
Entity of reduction	
From 10 mg to 5 mg	21/25 (18.1)
From 10 mg to 10 mg on alternate days	1/25 (0.9)
From 5 mg daily to 5 mg on alternate days	3/25 (2.7)
Response	
Complete response (CR)	1 (0.9)
Partial response (PR)	11 (9.5)
Stable of disease (SD)	85 (73.3)
Progressive disease (PD)	19 (16.3)
Toxicity	
No	33 (28.4)
Yes	83 (71.6)
Types of adverse reactions (G3‐G4) (%)	
Stomatitis and mucositis	5 (4.3)
Thrombocytopenia	5 (4.3)
Diarrhea	3 (2.6)
Metabolic adverse events (diabetes/hyperglycemia, dyslipidemia)	3 (2.6)
Fatigue	2 (1.7)
Pneumonitis	2 (1.7)
Anemia	1 (0.9)
Leukopenia with neutropenia	1 (0.9)
Skin rash/acne	1 (0.9)
Increased AST and/or ALT level	1 (0.9)
Hypertension	1 (0.9)

Everolimus administration was temporarily interrupted in 77 patients (66.4%), with a median delay of 28 days, while 25 subjects (21.6%) underwent dose reduction especially from 10 mg down to 5 mg.

Positive response was observed in 97 patients (83.7%) while 11 patients (9.5%) experienced a clinical benefit, that is, an improvement in symptoms and quality of life. Eighty‐three patients (71.6%) reported adverse effects while receiving study drug. The most common toxicities were stomatitis and mucositis, followed by hematological effects (grade 3 thrombocytopenia was observed in three patients who presented grade 1 thrombocytopenia at baseline, and grade 3 anemia occurred in 1 patient). Other reported events included diarrhea, fatigue, skin rash/acne, metabolic adverse events, and liver toxicity, and only one patient presented grade 3 hypertension. Grade 3 pneumonitis occurred in two patients and the adverse event was resolved by medical therapy with no *sequelae* in all cases.

Median PFS was 19 months (range: 3–71 months), while median OS was 44 months (range: 1–76 months).

**Figure 1:**
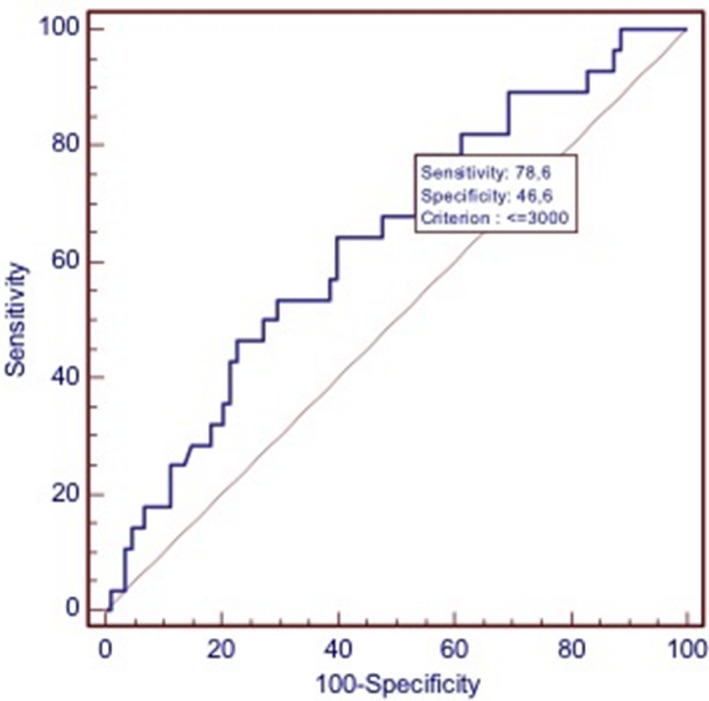
Receiver operating characteristics analysis based on everolimus cumulative dose (CD) with death as end point. In this model, sensitivity was 78.6% (95% CI: 59.0–91.7) and specificity was 46.6% (95% CI: 35.9–57.5). AUC was 0.642; *P* = 0.0124.

According to everolimus cumulative dose (CD), patients were stratified in two groups with CD ≤ 3000 mg (Group A) and CD > 3000 mg (Group B). This cut‐off, with the highest sensitivity and specificity for estimating the everolimus cumulative dose was set at 3000 mg after ROC curve analysis (Fig. 1). Groups A (*n* = 68) and B (*n* = 48) were homogeneous for main characteristics, including gender, age, grading, stage at initial diagnosis, response rate, grade 3–4 toxicity and all the patients received everolimus for at least 3 months.

Univariate analysis showed that line of therapy of everolimus (everolimus received as first line vs. everolimus received after 1 to 3 lines of chemotherapy, *P* = 0.045) and everolimus CD > 3000 mg (*P* = 0.04) were associated with a better OS.

At multivariate analysis, everolimus CD > 3000 mg resulted an independent prognostic factor both for OS [Hazard Ratio (HR): 0.16; 95% Confidence Interval (CI): 0.06–0.41, *P* < 0.0001] and for PFS (HR: 0.56; 95% CI: 0.34–0.92, *P* < 0.047).

**Figure 2:**
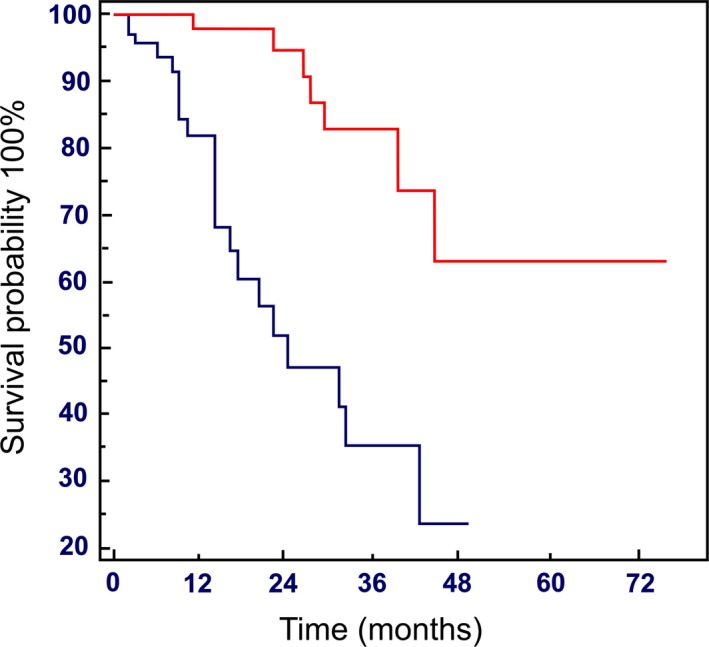
Overall survival stratified by the everolimus cumulative dose (CD): 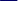 ≤3000 mg (Group A) and 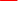 >3000 mg (Group B).

Although the rate of best response and grade 3–4 toxicity were comparable between the two groups, patients in group A experienced more dose modifications (delays or reductions according to medical decisions and patients’ preference) as compared with patients in group B. Median DI was 88.5% (range: 29.5–100%) in group A, while median DI was 96% in group B (range: 37–100%). Median OS was 24 months in Group A (range: 2–42 months), while in Group B it was not reached (HR: 26.9; 95% CI: 11.0–76.7; *P* < 0.0001) (Fig. 2).

**Figure 3:**
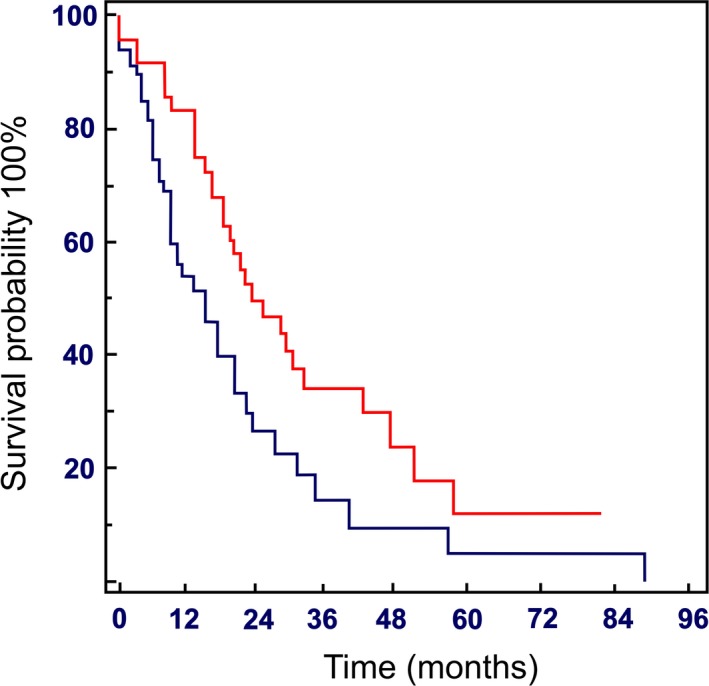
Progression‐free survival stratified by the everolimus cumulative dose (CD): 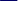 ≤3000 mg (Group A) and 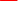 >3000 mg (Group B).

Furthermore, analysis of data showed that patients who maintained a DI higher than 9 mg/day experienced a significantly of longer OS and a trend to higher response rate, although not statistically significant. Median PFS was 15 months (range: 0–57 months) in Group A and 23 months (range: 0–71 months) in Group B, with a significant advantage for this latter group (HR: 1.85; 95% CI: 1.141–2.996; *P* = 0.0125) (Fig. 3).

Finally, patients were stratified into four groups:

1. neither dose reduction nor dose delay,
2. dose reduction only
3. dose delay only
4. dose reduction and dose delay.

The Kaplan–Meier analysis showed a trend toward better OS (*P* = 0.7401) in patients that experienced neither dose reduction nor dose delay (median OS not reached), followed by patients that experienced only dose delay (median OS not reached), than patients with only dose reduction (median OS = 124 months) and finally patients that experienced both dose reduction and dose delay (median OS = 92 months).

## Discussion

The aim of this study was to analyze clinical factors potentially influencing the global outcome of advanced PNET patients receiving everolimus in clinical practice in order to help clinicians in the decision‐making process for the identification of treatment strategy in this setting.

The PI3K/Akt/mTOR pathway has been proved to be involved in the development and progression of PNETs 9, 17 with a high prevalence of mutation regarding nearly all the members of this molecular pathway 18, 19, 20.

On this basis, everolimus has been shown to be an effective therapeutic agent in these tumors 21, 22 since Yao et al. reported a significant improvement in PFS in patients treated with the mammalian target of rapamycin compared with placebo. This led to the introduction of this drug in the treatment strategy for advanced PNETs 10.

In this study, we investigated the prognostic role of CD and DI of everolimus in advanced PNETs achieving a median OS of 24 months (range: 2–42 months) in group A (CD ≤ 3000 mg), while in group B (CD > 3000 mg) it was not reached (HR: 26.9; 95% CI: 11.0–76.7; *P* < 0.0001) (Fig. 2). Median PFS was 15 months (range: 0–57 months) and 23 months (range: 0–71 months), respectively, in group A and B, with a significant advantage for this latter group (HR: 1.85; 95% CI: 1.141;2.996; *P* = 0.0125) (Fig. 3).

Furthermore, analysis of data showed that patients who maintained a DI higher than 9 mg/day experienced a significantly of longer OS and a trend to higher response rate, although not statistically significant.

Finally, this study confirms the independent prognostic role of stage of the disease, showing also a correlation with tumor grading.

Many clinical trials documented drug‐related adverse events with everolimus, frequently leading to dose adjustments or treatment interruption 10, 23, therefore, lower CD and DI of everolimus are administrated, with potential negative effects on patients’ outcomes.

The safety profile of everolimus has been proved to be generally acceptable in PNET patients, with severe toxicities occurring only in a tiny minority of subjects 24. The onset of adverse events seems to be not correlated with the presence of liver metastasis, while previous treatment might affect the tolerability of this drug 25.

Furthermore, the onset of toxicities, especially mucositis, appear to be correlated with a major disease control rate (DCR) 24 and a longer PFS 26, as already known for other targeted agents used in the management of PNETs 27.

To the best of our knowledge, this study is the first to investigate the prognostic significance of CD and DI of everolimus in advanced PNETs.

Although the prolonged OS observed in patients with higher CD might be due to the fact that patients who maintain higher dose are usually better responsive to therapy, our results showed a significant correlation between CD and DI of everolimus and OS in a large series of patients with PNET, namely better prognosis in patients maintaining both higher CD and higher DI.

Furthermore, it is important to notice that patients in group B with a DI ≥ 9 mg/day presented a significantly in longer OS. This suggests that it could be more effective to maintain the full dose during the treatment, allowing temporarily interruptions when necessary. Patients that experienced only dose delay showed a trend to higher OS if compared with patients with only dose reduction and patients with both dose reduction and dose delay.

The difference in OS in patients treated with everolimus seemed strictly dependent upon the CD taken by sensitive patients, thus suggesting that we should make the best efforts in order to manage toxicity without interrupting the treatment.

Dose delays and dose reductions with regard to amount reduced and length of delay may not directly translate into quantitative reductions in therapy intensity, so it could surely be interesting to determine a significant cut‐off value for both dose delay and dose reduction. However, treatment duration was very heterogeneous among patients, and unavoidably conditioned by everolimus efficacy, making difficult to identify a significant cut‐off value.

Although selection bias and the retrospective nature of the study may have influenced our findings, overall present data seem to suggest that CD and DI potentially play a prognostic role for patients with advanced PNETs treated with everolimus.

This should prompt efforts to continue everolimus administration in responsive patients up to at least 3000 mg despite delays or temporary interruptions.

## Conflict of Interest

The Authors declare no conflicts of interest directly relevant to this study.
